# MiR-101: An Important Regulator of Gene Expression and Tumor Ecosystem

**DOI:** 10.3390/cancers14235861

**Published:** 2022-11-28

**Authors:** Ning Liu, Chunsheng Yang, Ang Gao, Meili Sun, Deguan Lv

**Affiliations:** 1 Department of Oncology, Central Hospital Affiliated to Shandong First Medical University, Jinan 250013, China; 2 UPMC Hillman Cancer Center, University of Pittsburgh, Pittsburgh, PA 15232, USA

**Keywords:** miR-101, microenvironment, suppressor, proliferation, migration, invasion, apoptosis, therapy

## Abstract

**Simple Summary:**

Abnormal expression of miRNA is often observed in cancer. MiR-101 is closely associated with tumor initiation and progression. In this review, we summarized new findings regarding the role of miR-101 in cancer and the potential mechanisms of targeted gene degradation and microenvironmental regulation.

**Abstract:**

MiRNAs are small single-stranded non-coding RNAs. MiRNA contributes to the transcriptional and post-transcriptional regulation of mRNA in different cell types, including mRNA transcription inhibition and mRNA decay and phenotypes via the effect of several essential oncogenic processes and tumor microenvironment. MiR-101 is a highly conserved miRNA that was found to alter the expression in various human cancers. MiR-101 has been reported to have tumor oncogenic and suppressive effects to regulate tumorigenesis and tumor progression. In this review, we summarize the new findings about the roles of miR-101 in cancers and the underlying mechanisms of targeting genes degradation and microenvironment regulation, which will improve biological understanding and design of novel therapeutics.

## 1. Introduction

MiRNAs were found in 1993 by Lee et al. [1], who identified a class of small molecule RNAs named Lin-4 [1], and the discovery of this new biomolecule provides a new way of thinking about the regulation of cell fate in physiology and pathophysiology in eukaryotes. The latest released miRBase database (v22) contains 48,860 mature miRNAs, of which 2654 miRNAs are human miRNAs [2].

MiRNAs are a class of small (18–25 nucleotides) non-coding single-stranded RNAs [3,4]. Studies have revealed that miRNAs share common biologic characteristics, such as evolutionarily conservation, stage, and tissue-specific expression. The mature miRNA can recognize specific target mRNA sequences utilizing the Watson–Crick base pairing principle, which ultimately leads to the degradation or translation inhibition of target mRNA, thus regulating the expression of target genes at the transcriptional or post-transcriptional level [5]. More than 5300 human genes, which represent 30% of all known genes, are regulated by miRNAs.

In cancers, as negative regulatory factors of gene expression, miRNAs regulate tumorigenesis and cancer progression by affecting numerous biological signaling pathways [6,7,8,9,10,11]. 

MiR-101 is highly conserved in various species. There are two gene loci producing miR-101 in humans, which are located in regions 1p31.3 and 9p24.1. MiR-101 contains two precursor RNAs: miR-101-1 and miR-101-2, with length of 75 bp and 79 bp, respectively [12]. MiR-101-3p is one of the most dominant members of mature miR-101, which is named because the dicer enzyme binds to the 3‘arm of pre-miR-101. 

Recent studies have shown that miRNA-101 plays important regulatory roles in the occurrence and development of fibrosis in pulmonary or liver, cardiovascular and cerebrovascular diseases, cancer, and other diseases. MiR-101 has become the focus and hotspot of cancer research [13,14]. Wang et al. [15] summarized the function of miR-101 in cancer cell proliferation, migration, and apoptosis via SRY-box transcription factor 9 (SOX9), enhancer of zeste 2 polycomb repressive complex 2 subunit (EZH2), Rac family small GTPase 1 (Rac1) etc. in 2018 [15]. Since then, a lot of new findings have been published. In this review, we review ongoing efforts to understand the roles of miR-101 in tumor development and progression and discuss the challenges and future steps to clarify the conflicting results of miR-101 as an oncogene and tumor suppressor, which provide approaches to leverage these discoveries for translational studies. 

## 2. MiR-101 Regulates Cancer Growth, Metastasis, and Therapeutic Resistance via Several Essential Oncogenic Processes

As described above, the genes producing miR-101 are in the 1p31.3 region and 9p24.1 region of the human genome. By using genomic quantitative polymerase chain reaction, Sooryanarayana et al. [16] found that 1p31.3 region loss is a common phenomenon in many cancers, such as breast, gastric, and prostate cancers. Further, they observed genomic losses of one or both miR-101 loci in a subset of glioblastoma multiforme, lung adenocarcinoma, and acute lymphocytic leukemia, which resulted in a deficiency of miR-101 expression in cancer tissues [16]. The downregulated miR-101 is proven to play important roles in cancer cell phenotype regulation, including cell proliferation, apoptosis, migration, invasion, and therapy resistance [17,18]. However, certain research finds that miR-101 affects tumor development as an oncogene, especially the in vivo studies. It was reported that increased miR-101 level predicts poor overall survival and disease-free survival in ovarian cancer patients [19]. 

### 2.1. The Role of miR-101 in Cell Proliferation

One of the cancer hallmarks is disruptions of negative feedback mechanisms that attenuate proliferative signaling and the consequent malignant growth of tumor cells. MiR-101 expression deficiency impairs negative feedback of proliferative signaling. Previous studies have confirmed that miR-101 inhibits cell proliferation in multiple tumors, and it exists as a tumor suppressor by affecting several essential oncogenic pathways. 

Consistent with previous studies, the discoveries also confirm that miR-101 expression is decreased in a wide variety of cancers [20]. The decreased miR-101 promotes cancer cell proliferation by increasing the expression of numerous oncogenes. In hepatocellular carcinoma, miR-101 is proven to specifically bind to vascular endothelial growth factor (VEGF) mRNA three prime untranslated region (3′-UTR) to decrease protein levels of VEGFA (one of the main isotypes of VEGF) [21] and inhibit VEGFR2 signaling pathway thereby impair the malignant behavior of HCC cells. MiR-101 could also inhibit the proliferation of liver cancer cells by targeting zinc finger protein 217 (ZNF217), mitogen-activated protein kinases (MAPK)/extracellular signal-regulated kinase (ERK) signaling pathways, and hepatocyte growth factor (HGF)/c-MET axis [22,23,24]. The expression level of miR-101 is decreased in liver cancer which loses the inhibition effect on the above target genes acting as on–off switches of particular mRNAs or by modulating the relationship between effector and target mRNAs. Similarly, miR-101 was found to regulate downstream targets of growth factor signaling in other cancers. MiR-101 inhibits the proliferation of gastric cancer cells by regulating the expression of proto-oncogene serine/threonine protein kinase (PIM 1) and AMP-activated protein kinase (AMPK). Mechanistically, the expression of AMPK is regulated by lncRNA SPRY4-IT1 via sponging miR-101 [25,26]. In melanoma, miR-101 regulated by lncRNA SNHG6 is negatively correlated with Ras-related protein Rap-2b (RAP2B) expression, which is a member of the *RAS* oncogene family. RAP2B overexpression reversed the inhibitory effect on melanoma cell proliferation induced by miR-101 [27]. MiR-101 is downregulated in NSCLC tissues, and overexpression of miR-101 inhibits the proliferation of NSCLC cells by targeting several essential proliferative genes, including ATP binding cassette subfamily C member 1 (ABCC1), zinc finger E-Box binding homeobox 1 (ZEB1), chromodomain Y-like (CDYL), myeloid cell leukemia-1 (Mcl-1) and metastasis-associated lung adenocarcinoma transcript 1 (MALAT-1) [28,29,30,31,32]. MiR-101 suppression also accelerates cervical cancer cell proliferation by promoting the expression of methionine adenosyltransferase II Alpha (MAT2A) [33]. Med19 enhances breast cancer cell proliferation, which is negatively correlated with miR-101 expression [34]. Furthermore, miR-101 mimics can reduce the proliferation rate of breast cancer cells by reducing the mRNA level of nuclear factor (erythroid-derived 2)-like 2 (Nrf2) [35]. In colon cancer cell lines, silencing of stanniocalcin 1 (STC1) reverses the tumor-promoting effects of miR-101 down-regulation [36], and overexpression of miR-101 generates anti-tumor effects by targeting the cAMP-responsive element binding protein 1 (CREB1) and Notch signaling pathway [37,38]. In pancreatic cancer, miR-101 inhibits cell proliferation by targeting STMN1 [39]. It was also announced that miR-101 reduces the expression of other *RAS* oncogenic family members, such as Rap1A, in prostate cancer and thereby inhibits cell proliferation, migration, and invasion [40]. Additionally, miR-101 inhibits the proliferation of retinoblastoma cells by targeting EZH2 and histone deacetylase 9 (HDAC9) [41]. Although many new target genes of miR-101 were identified, how miR-101 regulates these genes is not fully understood. It is important to clarify the intracellular mechanisms associated with miR-101 regulation as we harness this knowledge for therapeutic benefits.

MiR-101 regulates proliferation by affecting metabolism. CDGSH iron sulfur domain 1(CISD1) plays a key role in regulating maximal electron transport capacity and oxidative phosphorylation. CISD1 is up-regulated in lung adenocarcinoma, and CISD1 knockdown is noted to significantly inhibit lung adenocarcinoma cell proliferation. The miR-101 is identified to be upstream of CISD1 [42]. MiR-101 disrupts the transcription of mitochondrial DNA (mtDNA) via down-regulating mitochondrial transcription initiation complex proteins transcription factor B2, mitochondrial (TFB2M), and mitofilin to inhibit cancer cell proliferation in osteosarcoma [43]. MiR-101 directly interacted with the 3′-UTR of KRAS mRNA to weaken glycolytic metabolism in NSCLC [44]. Overexpression of miR-101 could inhibit the growth of liver cancer stem cells xenograft tumors, which works through targeting annexin A2 (ANXA2) [45]. 

Studies also revealed that miR-101 could control proliferation by affecting the protein degradation process. Ubiquitin-specific protease 47 is a deubiquitinating enzyme that removes ubiquitin conjugates from their substrates, thereby altering their stabilities, localizations, or activities. MiR-101 regulates ribosomal protein L11 (RPL11) localization and its interaction with MDM2pProto-oncogene (MDM2) by inhibiting the ubiquitin-specific peptidase 47 (USP47)-induced deubiquitylation of RPL11. MiR-101 was found to bind directly to the 3′-UTR region of the USP47 mRNA and inhibit its expression [46]. Cullin 4B (CUL4B) encodes a scaffold protein that organizes the cullin-RING (an interesting new gene) ubiquitin ligase (E3) complex during ubiquitylation. In non-small cell lung cancer and prostate cancer, CUL4B is confirmed as a functional target of miR-101, and its knockdown results in a strong alleviation of cell proliferation, which is enhanced by the silencing of miR-101 [47,48]. MiR-101 may regulate protein intracellular distribution and decay. MiR-101 expression is low in cervical squamous cell carcinoma, while importin karyopherin subunit alpha 2 (KPNA2) expression is high MiR-101 suppresses the progression of cervical squamous cell carcinoma by targeting and down-regulating KPNA2 [49]. 

Together, miR-101 inhibits tumor cell proliferation by targeting many essential oncogenic targets. However, some controversial studies have shown that miR-101 promotes cell growth and acts as an oncogene. Myeloid-derived suppressor cells (MDSCs) are important cellular components in the tumor microenvironment which protect tumor cells from the attack of the immune system and promote tumor progression [50]. Cui et al. show that MDSCs stimulate the expression of miR-101 in ovarian cancer cells by targeting C-terminal binding protein 2 (CTBP2) and promoting the formation of tumor stem cells [19]. Therefore, it is necessary to further study whether the controversial discovery is caused by tissue specificity, tumor grade, subtype, or other reasons.

### 2.2. The Role of miR-101 in Migration

MiR-101 has been identified as a key regulator in several stages of metastasis. Epigenetic changes, particularly histone and DNA methylation, play an important role in tumor progression, especially metastasis. Trimethylation refers to the methylation of histone H3K9me3, H3K27me3, and H3K79me3, which can inhibit transcription from regulating phenotype, in which H3K27me3 is the most frequent modification that regulates transcription [51]. Enhancer of zeste homolog 2 (EZH2) is an essential nuclear transcription regulator that encodes the core catalytic subunit of polycomb polysive complex 2 (PRC2) that directly trimethylates the 27th lysine residue of histone 3. EZH2 promotes cell migration, thus promoting tumor progression. Studies have demonstrated that miR-101 negatively regulates EZH2 expression in breast cancer, prostate cancer, and glioma [52,53,54]. Huang et al. revealed two-way negative feedback between EZH2 and miR-101. The imbalance of this negative feedback pathway continuously increases the expression level of EZH2, thus promoting the proliferation of tumor cells [55]. In bladder cancer cells, lncRNA SPRY4-IT1 could directly interact with miR-101 acting as a miRNA sponge, and miR-101 inhibition leads to increased EZH2 expression. EZH2 inhibits the E-cadherin promoter activity through methylating of H3K27me3 and promotes bladder cancer cell invasion and metastasis [56]. Methylation in the gene promoter locus is catalyzed by DNA methyltransferases (DNMTs). DNMT3A (DNA Methyltransferase 3 Alpha) is a de novo methyltransferase that can methylate 5-methylcytosine methylation [57]. Wei et al. [58] found that miR-101 directly inhibited the mRNA expression and protein level of DNMT3A by complementary binding with the 3′-UTR region, leading to increased methylation level in the promoter regions of tumor suppressor genes, thus suppressing tumor growth and invasion, but the miR-101 expression was frequently down-regulated in HBV-related HCC tissues [58]. The negative correlation between miR-101 and DNMT3A expression has been confirmed in breast cancer, lung cancer, brain glioma, and other tumors [15]. MiR-101 has been proven to inhibit cell migration and invasion by inhibiting the expression of DNMT3A and up-regulating the expression of E-cadherin in breast cancer cells [59]. MiR-101 is also closely related to the prognosis of glioma. Low miR-101 expression informs poor prognosis in glioma patients. MiR-101 inhibits the expression of EZH2 and DNMT3A, which leads to increased levels of H3K4me3 and H4K20me3 in the core promoter region of LIM domain only 3 (LMO3), thereby inhibiting the expression of LMO3 and metastasis of glioma cells [60].

Except for EZH2 and DNMT3A, attenuation of miR-101 is also required for other genes expression and metastasis. Ectopic overexpression of miR-101 suppressed cancer-associated fibroblasts (CAFs) activation and abrogated the promoting effect of CAFs on migration and invasion of non-small cell lung cancer cells (NSCLC) through attenuating CAFs’ effect on epithelial–mesenchymal transition (EMT) process, metastasis-related genes matrix metallopeptidase 9 (MMP9), twist family BHLH transcription factor 1(TWIST1-), and AKT/endothelial nitric oxide synthase (eNOS) signaling pathway. Further study indicates that vascular endothelial growth factor A (VEGFA) is a novel target of miR-101, and CAFs-derived VEGFA mediates the effect of miR-101 on migration and invasion of lung cancer cells [61], suggesting a new combination of VEGF and miR-101 for metastasis therapy. Ceramide synthesis by Ceramide Synthase 6 (CERS6) is required for cell migration and metastasis in lung cancer. MiR-101 can reduce CERS6 expression in lung cancer [62]. In both cervical cancer and NSCLC, miR-101 inhibits metastasis by inhibiting CXCL6 expression [63,64]. Moreover, in NSCLC cells, miR-101 also shows an inhibitory effect on migration by targeting CUL4B, CISD1, MALAT-1, and DNMT3A [32,42,47,65]. In papillary thyroid carcinoma (PTC), CLDN1 is the target of miR-101, and overexpression of CLDN1 can reverse the inhibitory effect of miR-101 on cell migration and invasion [66]. MiR-101 could also suppress colorectal cancer cell migration by negatively regulating Rap1b [67]. The previous description mentioned that the expression level of miR-101 declines in hepatocellular carcinoma, and miR-101 suppresses migration by targeting the HGF/c-Met pathway [23]. MiR-101 could inhibit metastasis of liver cancer, and it was downregulated in liver cancer stem cells. ANXA2 is a novel target of miR-101, which mediates the role of miR-101 in the migration and invasion ability of liver cancer stem cells [45]. Additionally, miR-101 is downregulated in colorectal cancer tissues, lower expression of miR-101 results in overexpression of ZEB1, which promotes the migration of colorectal cancer cells [68]. MiR-101 is discovered to repress cell metastasis of ovarian cancer via decreasing Fibronectin 1(FN1) expression [69]. It is also found that downregulation of miR-101 increases the transmigration of breast cancer cells through the brain endothelium in vitro by inducing prostaglandin-endoperoxide synthase-2 (PTGS2, also known as COX-2), expression in cancer cells and miR-101 mediates its effect by modulating COX-2-MMP1 (Matrix Metalloproteinase-1) signaling pathway [70]. Like other cancers, the expression of miR-101 is significantly reduced in bladder cancer tissue compared with that in adjacent non-tumor tissue, and miR-101 can inhibit bladder cancer cell migration and invasion via directly targeting frizzled class receptor 4 (FZD4) [71]. Additionally, it was recognized that miR-101 could suppress the expression of tripartite motif containing 44 (TRIM44) by directly targeting its 3′-UTR, thereby reducing proliferation, migration, and invasion of glioblastoma cells [72]. Those studies support the use of miR-101 restoration treatment for aggressive cancers. 

### 2.3. MiR-101 and Invasion

Tumor invasion is considered one of the prime reasons for tumor progression. Epithelial–mesenchymal transformation is a biological process that allows cancers to gain metastatic capacity. E-cadherin is a key molecule of EMT. Down-regulation of E-Cadherin is a typical hallmark during EMT in cancer progression. Down-regulation or inhibition of E-cadherin leads to the release of β-catenin, which can be transferred into the nucleus and acts as a transcriptional activator to initiate EMT. MiR-101 up-regulates the expression of E-cadherin and inhibits EMT [56,73]. Zeb1/2 binds to the E-box in the E-cadherin promoter region and acts as a transcriptional repressor to inhibit the expression of E-cadherin [74,75]. Guo et al. found miR-101 directly binds to the seed sequence on the 3′-UTR of ZEB1 or ZEB2, resulting in the inhibition of ZEB1 and ZEB2 expression and thus inhibiting EMT [76]. Kailash et al. also confirmed that miR-101 inhibits the expression of ZEB1, ZEB2, *RAS* homolog family member A(RhoA), and Rac family small GTPase 1 (RAC1), and a significant negative correlation between miR-101 expression and lymph node metastasis was confirmed in clinical specimens [77]. Additionally, miR-101 is significantly down-regulated in osteosarcoma and could inhibit the proliferation and invasion of osteosarcoma by targeting ZEB2 [78]. It was recognized that miR-101 could suppress the invasion ability of cervical cancer cells by inhibiting the expression of MAT2A [33]. Furthermore, miR-101 could inhibit the invasion of NSCLC cells by targeting CDYL [30]. It was also elucidated that miR-101 suppresses cell invasion and proliferation in gastric cancer by targeting PIM-1 and serum response factor (SRF) [25,79]. As described above, the expression level of miR-101 was decreased and acted as a negative regulator of ZNF217 in liver cancer, which could not only suppress proliferation but also inhibit the invasion of liver cancer cells [24]. Moreover, miR-101 was found to suppress the invasion of hepatocellular carcinoma cells by targeting VEGF-C (another member of the VEGF family) and Girdin [80,81]. These studies support the importance of miR-101 loss in tumor metastasis.

### 2.4. MiR-101 in Apoptosis

In addition to affecting the proliferation, migration, and invasion of tumor cells, miR-101 is also involved in the regulation of programmed cell death, such as apoptosis in a variety of tumors. Wang et al. summarized the function of miR-101 in apoptosis via SOX2, a mechanistic target of rapamycin kinase (mTOR), VEGF-2, etc., in 2018 [15]. Since then, many new target genes have been identified. In oral cancer, the increased level of miR-101 promotes apoptosis by suppressing BicC family RNA binding protein 1 (BICC1) [82]. MiR-101 could promote cell apoptosis in medulloblastoma targeting forkhead box P4 (FOXP4) and EZH2 [83]. Furthermore, in diffuse large B cell lymphoma (DLBCL), miR-101 regulates cell apoptosis by targeting lysine demethylase 1A (KDM1A) and MAPK kinase 1 (MEK1). This reveals that miR-101 may be a useful tool for the treatment of DLBCL patients [84,85]. Additionally, miR-101 also promotes cell apoptosis by inhibiting the *RAS*/Raf/MEK/ERK signaling pathway in nasopharyngeal carcinoma [86]. MiR-101 also has a pro-apoptotic role in breast cancer via Janus Kinase 2 (JAK2) [11]. Zhu et al. revealed overexpression of miR-101 promotes TRAIL-induced mitochondrial apoptosis in papillary thyroid carcinoma by targeting c-met and MCL-1 [87]. MiR-101 ‘drug’ represents a promise for cancer therapy.

### 2.5. miR-101 and Tumor Chemoresistance

Chemotherapy is one of the main options for cancer patients’ therapy. Patients no longer benefit from chemotherapy because of drug resistance. MiR-101 is involved in the regulation of drug resistance in a variety of cancers. MiR-101 affects the sensitivity of tumor cells to chemotherapy drugs by regulating relevant processes, including drug transporters, apoptosis regulation, cell cycle regulation, autophagy, etc.

In 2009, researchers from Scripps Research Institute discovered a protein called glycoprotein, P-gp (P-Glycoprotein). This protein blocks many chemotherapeutic drugs from entering cells, which is one of the main reasons cancer cells become resistant to them. However, overexpression of miR-101 leads to decreased P-gp expression in gastric cancer cells and enhances the sensitivity of tumor cells to cisplatin or vincristine [88]. Chai et al. confirm that up-regulated miR-101 can also increase the sensitivity of liver cancer to cisplatin by negatively regulating DNA-PKCs/Akt/NF-κB signals [89]. Negative regulation of EZH2 by miR-101 can enhance the sensitivity of bladder urothelial carcinoma to cisplatin [90]. Long non-coding RNA MALAT1 promoted the chemoresistance to temozolomide by suppressing the miR-101 signaling pathway via directly binding it in glioblastoma cells [91]. MiR-101 acts as a tumor suppressor in HER2-positive breast cancer cells, improving targeted therapy of lapatinib and trastuzumab [92].

Autophagy is a highly conserved cell metabolic process that plays a dual role in tumor procession, including therapy resistance. Studies have shown that autophagy protects tumor cells from the attack of chemotherapeutic drugs by cleaning up large molecules or organelles damaged by chemotherapy and ionizing radiation. MiR-101 enhances cisplatin-induced apoptosis of HepG2 cells by inhibiting autophagy of targets such as member *RAS* oncogene family (RAB5A), STMN1, and autophagy-related 4D cysteine peptidase (AtG4D) in HCC [93]. Inhibition of miR-101 expression can increase the expression of autophagy-related proteins such as Rab5a and Atg4d and enhance the resistance of pancreatic ductal adenocarcinoma (PDCA) to gemcitabine [94]. MiR-101 can block autophagy-characterized acidic vesicle organelles (AVOs) and enhance the sensitivity of osteosarcoma cells U-2OS to doxorubicin [95]. Re-expressing miR-101 has great promise for cancer therapy. As the therapeutic potential of miR-101 is explored, the combination of miR-101 and chemical therapy will probably follow.

### 2.6. miR-101 and Radiosensitivity

Radiation therapy is another important available approach for tumor therapy, which kills tumor cells by inducing double-strand DNA breaks (DSBs). Some tumors, such as nasopharynx cancer, can be cured by radiation therapy. However, radiation resistance also occurs in tumor development, and effective repair of tumor cells is a key factor hindering the success of radiation therapy. DNA double-strand breaks induced by radiation can be repaired by non-homologous end-source ligation repair (NHEJ) and homologous recombination repair (HRR). Cells lacking AT mutated (ATM) enzyme—a member of the phophatidylinositol-3-OH kinase (PI(3)K) family—are sensitive to ionizing radiation (IR) due to inefficient DNA DSB repair [96]. It has been proved in lung cancer cell lines and glioblastoma cell lines that miR-101 can bind to the 3′-UTR of DNA-PKCs or ATM mRNA to negatively regulate DNA-PKCs and ATM mRNA. Upregulation of miR-101 can reduce the protein levels of DNA-PKCs and ATM, which leads to acquiring radiosensitivity in tumor cells to radiation [97]. Chen et al. confirmed that miR-101 could sensitize HCC cells through negative modulation of WEE1 G2 checkpoint kinase (WEE1) [98].

Ectopic expression of miR-101 can increase the radiosensitivity of nasopharyngeal carcinoma cells by targeting STMN1 inhibition [99]. MiR-101 can also negatively regulate the mTOR signaling pathway to increase the radiosensitivity of A549 cells [100]. Decreased expression of miR-101 can increase the expression levels of downstream target proteins ATM and mTOR and increase the radiation resistance of esophageal squamous cell carcinoma [101]. In lung adenocarcinoma cells, miR-101 can enhance LUAD cell sensitivity to radiotherapy by regulating the expression of baculoviral IAP repeat containing 5 (BIRC5) [102]. These data suggest that the loss of miR-101 plays a significant role in cancer therapy, showing potential value as a therapeutic tool in the future. The advancement of such studies will help to guide the development of miR-101-based treatment for sensitization to radiotherapy.

**Figure 1:**
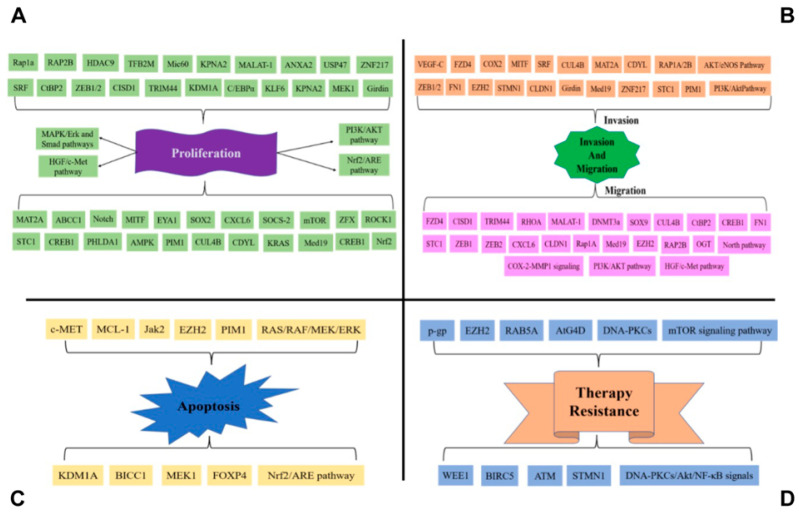
MiR-101 regulates proliferation, migration, invasion, apoptosis, and therapy resistance via regulates numerous targets. (**A**) MiR-101 targets important oncogenes such as SOX2 and AKT to suppress tumor growth. (**B**) MiR-101 directly targets the mRNAs encoding the ZEB1 and ZEB2 et al. in cancers; as such, when miR-101 level is reduced, the expression of ZEB1 et al. becomes elevated, which suppresses the expression of epithelial genes to promote the epithelial–mesenchymal transition (EMT) to promote cancer cell local invasion, migration, and metastatic tumors. (**C**) MiR-101 targets apoptosis-related genes MCL-1 et al. to promote cancer cell apoptosis. (**D**) MiR-101 affects therapy resistance in cancers via NF-kb signaling and other pathways.

Overall, the miR-101 regulates proliferation, migration, invasion, apoptosis, and therapy resistance via regulates numerous targets (Figure 1) in transcriptional and post-transcriptional manners (Table 1).

## 3. MiR-101 Inhibits Cancer by Remodeling the Tumor Ecosystem

Tumor microenvironment refers to the occurrence, growth, and metastasis of tumors and the internal and external environment in which tumor cells are located. Tumor cells and tumor microenvironments are closely related. Tumor cells can improve their developmental conditions through autocrine and paracrine promotion. Systemic and local tissues can also limit and affect the development of tumors through immune secretion changes.

### 3.1. Crosstalk between MiR-101 and Microenvironment

Interactions between cancer cells and their microenvironment can greatly influence tumor progression and metastasis. Previous studies confirmed that miR-101 inhibited the growth of breast cancer cell line MCF-7 in the presence of estrogen in the medium, but it promotes cell growth in the estradiol-free medium [121]. This suggests that miR-101 may inhibit breast cancer growth by affecting estrogen secretion. However, we still do not know whether the regulation is causal. 

Hypoxia is present in many malignant tissues. The hypoxic environment increases the expression of miR-101 in human umbilical vein endothelial cells (HUVECs), astrocytes, HeLa, and U937, which is dependent on HIF-α expression [122]. Similarly, our research demonstrates that hypoxia promotes the expression of miR-101 in breast cancer cells [123]. This evidence shows that the microenvironment regulates tumor progression mediates by miR-101. Understanding these interactions is important for the therapies development in cancers.

### 3.2. MiR-101 and Tumor Angiogenesis

The typical manifestation of early tumors is little or no vascularization. As the tumor grows, tumor cells stimulate the secretion of vascular endothelial growth factor and angiogenesis to meet the needs of nutrients. 

To date, the role of miR-101 in angiogenesis is contradictory. VEGF is one of the most essential factors in angiogenesis. MiR101 is known as a key regulator of angiogenesis via post-transcriptional regulation of VEGF. It has also been confirmed that miR-101 inhibits angiogenesis in gastric cancer by down-regulating the expression of VEGF-C [124]. By using the liver cancer model, Wang et al. found that overexpression of miR-101 could significantly reduce the level of VEGF, and further studies confirmed that miR-101 affected the secretion of VEGF by inhibiting the expression of junB proto-oncogene/AP-1 transcription factor subunit (JunB) [73]. MiR-101 was found to target VEGF mRNA 3′-UTR to regulate its expression in hepatocellular carcinoma [21]. Moreover, the VEGF mRNA is also identified as the target of miR-101 in cholangiocarcinoma cells. MiR-101 could inhibit VEGF or COX-2 expression by directly targeting the 3′-UTR of VEGF or COX-2 mRNA by which inhibits angiogenesis [125]. 

However, in breast cancer cells, we found that miR-101 can target and down-regulate VHL expression, which is a negative regulator of hypoxia-inducible factor 1 subunit alpha (HIF1α) to increase HIF-1α expression and HIF-1α downstream target VEGFA expression. MiR-101 could promote angiogenesis by indirectly promoting VEGFA gene expression [21]. As described above, miR-101 can improve blood perfusion in the ischemic hind limbs of mice, confirming that hypoxia-induced miR-101 plays an important role in angiogenesis and vascular remodeling after ischemia [122]. As mentioned above, hypoxia induces miR-101 expression in multiple cancers. Therefore, the relationship between miR-101 and angiogenesis needs to be further studied.

### 3.3. miR-101 and Tumor Immunology

Different from the roles in proliferation, migration etc. in vitro, miR-101 may play oncogenic and suppressive roles in vivo via regulating the immune response. However, this is still new research filed for miR-101.

MiR-101 regulates the differentiation of immune cells. Tumor-associated macrophages (TAMs) are related to tumor development [126]. Currently, TAMs have become valuable targets for cancer therapy. TAMs are divided into two phenotypes based on their activation status and function, M1 and M2. M1 acts as a tumor suppressor, while M2 plays a protumorigenic role in tumor development and progression. Zhao et al. revealed that overexpression of miR-101 in M1 could induce M1-to-M2 macrophage-type conversion, which leads to promoting cell proliferation and migration of breast and ovarian cancer cells by inhibiting CCAAT/enhancer-binding protein (C/EBP)α, and kruppel-like factor 6 (KLF6) expression [127]. MiR-101 binds to the 3′-UTR of tribbles pseudokinase 1 (TRIB1) mRNA, leading to increased transcription and secretion of interleukin-8 to regulate macrophage 2 differentiation in prostate cancer and control the inflammatory profile of human primary macrophages and prostate cancer cells [128]. Wu et al. suggest that miR-101, targeting dual specificity phosphatase 1 (DUSP1), regulates MAPKs activation during sorafenib-mediated inhibition of macrophage-induced hepatocarcinoma growth [129]. Gao also found that miR-101 affects macrophage polarization and might be an important factor associated with HCC prognosis and immune infiltration [130]. MiR-101 also could regulate the innate immune responses of macrophages to LPS by targeting MAPK phosphatase-1 (MKP-1) [12]. Yasuo Takashima et al. revealed that in primary central nervous system lymphoma (PCNSL), miR-101 regulates Th-1/Th-2 status, Treg status, and immune checkpoints [131]. 

**Figure 2:**
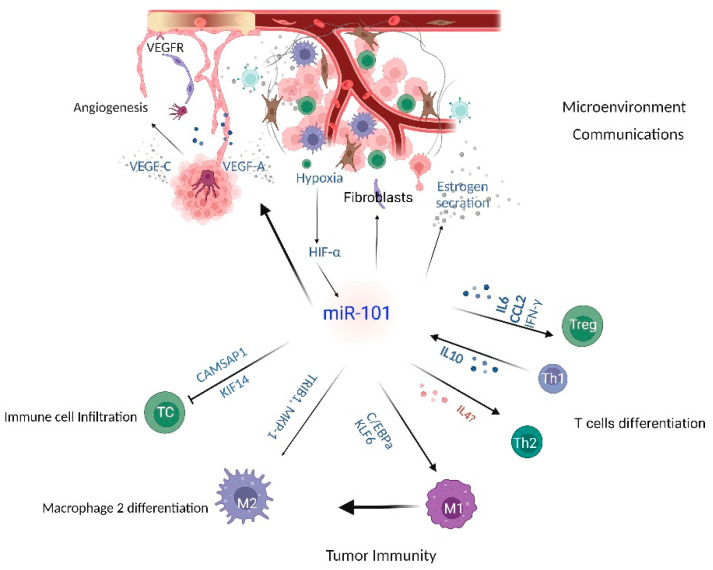
MiR-101 in context: tumor microenvironmental inputs.

Emerged evidence indicated that miR-101 might promote tumorigenesis by suppressing immune infiltration and promoting immune escape. Kinesin family member 14 (KIF14) was up-regulated in a variety of cancers, including lung adenocarcinoma. GSEC/TYMSOS inhibited miR-101-3P to increase KIF14 expression in lung adenocarcinoma to promote immune checkpoint-related gene expression, immune cell biomarkers, and tumor immune cell infiltration [132]. Calmodulin-regulated spectrin-associated protein 1 (*CAMSAP1*) suppresses immune cell infiltration in liver hepatocellular carcinoma, and the LINC01748-miR-101-3p axis is specifically responsible for *CAMSAP1* overexpression [133]. Furthermore, M2-polarized tumor-associated macrophages transfer miR-101 to cancer cells to increase ZEB1 and PD-L1 expression, thereby stimulating the immune escape of ovarian cancer cells [134]. MiR-101 is considered one of the useful markers for cancer immunotherapy (Figure 2).

## 4. Conclusions

Cancer-associated change in miR-101 expression patterns is emerging as promising diagnostic markers that often correlate with histological stage, tumor progression, and patient survival [34,56,88,94,98,125,135,136,137,138,139]. MiR-101 has key roles in cancer initiation, progression, and metastasis as miR-101 is a critical regulator of gene expression by binding to complementary sequences in the 3′-UTR of mRNAs to target them for degradation or prevent the target gene translation. Genetic loss of tumor suppressor miR-101 is associated with human cancers via affecting a lot of signaling pathways in cancers.

Although miR-101 can have either an oncogenic or tumor-suppressive role, research has shown that miRNA expression is globally suppressed in tumor cells compared with normal tissue, suggesting that miR-101 may be a suppressor [140]. Therefore, it is important to figure out whether miR-101 is an oncogene or suppressor, and it is essential to clarify whether these contradictory results are caused by methodology and technology. However, it is interesting that miR-101 is proven to be a tumor suppressor in vitro studies, while more and more in vivo results show that miR-101 promotes tumor progression. A key factor in vivo is the tumor microenvironment, especially the presence of immune cells. In addition to genomic alterations, hypermethylation of CpG islands at the gene promoters of tumor-suppressive miRNAs, disruption of miRNA production by depletion of any of the miRNA processing factors [141], the miR-101 secretion by exosome may aggravate the deficiency of miR-101 in cancer cells. Moreover, the secreted miR-101 inhibits immune response [142,143], such as Th-1/2 differentiation and killer T cell infiltration. A question may arise regarding how tumor cells survive in the secreted miR-101 microenvironment. The cancer stem cell, responsible for maintaining tumor heterogeneity, fueling tumor growth, and therapy resistance, may be one reason. Research reports that miR-101 promotes ovarian stem cell self-renewal and proliferation [19]. MiR-101 is closely related to the tumor microenvironment in a two-way communication manner. On the one hand, oxygen is essential for tumor growth and metastasis. Hypoxia can increase miR-101 expression in tumor cells. On the other hand, miR-101 stimulates angiogenesis by activating the HO-1/VEGF/eNOS axis via Cul3 targeting. In addition to indirectly participating in angiogenesis, miR-101 can also directly participate in the regulation of angiogenesis in tumor tissues by binding with the 3UTR of VEGF mRNA. 

Overall, in this review, we summarized new findings regarding the role of miR-101 in cancer and the potential mechanisms of targeted gene degradation and microenvironmental regulation, which will bring insight into miR-101 research and cancer treatment. Consequently, the roles of miR-101 in cancer and the underlying mechanisms are still needed to be further explored to better reveal its clinical potential.

## Figures and Tables

**Table 1:** New target genes and dysregulation of miR-101 in proliferation, apoptosis, angiogenesis, drug resistance.

Disease	Target Genes	References	Participation
Cervical Cancer	ZEB1	Fan, M.J., et al. [103].	EMT
	CXCL6	Shen, W., et al. [63].	Growth/metastasis
	MAT2A	Chen, H.F., et al. [33].	Proliferation/invasion
	KPNA2	Wang, H., et al. [49].	Proliferation
	COX-2	Liu, Y., et al. [104].	Angiogenesis
NSCLC	mTOR-signaling pathway	Li, Z.H., et al. [100].	Irradiation
	MCL1	Wang, H.Q., et al. [105].	Drug resistance
	ABCC1	Shao, N., et al. [28].	Proliferation/Drug resistance
	CUL4B	Xie, F.W., et al. [47].	Proliferation/migration/invasion
	ZEB1	Han, L., et al. [29].	Proliferation/metastasis
	CDYL	Li, K., et al. [30].	Proliferation/invasion
	KRAS	Ding, C.Z., et al. [44].	Proliferation/glycolysis
	SOX9	Kong, X.J., et al. [106].	Progression
	CUL4B	Zhang, H.F., et al. [107].	progression
	Mcl-1	Shahverdi, et al. [31].	Proliferation/apoptosis
	CISD1	Jiang, X.L., et al. [42].	Proliferation/migration
	USP47	Park, et al. [46].	Proliferation
	MALAT-1	Zhang, X.Q., et al. [32].	Proliferation/metastasis
	DNMT3a	Yan, F., et al. [65].	Migration
Ovarian Cancer	ZEB1	Liang, H.H., et al. [108].	Invasion/metastasis
	ZEB1/ZEB2	Guo, F., et al. [76].	EMT
	FN1	Liang, H.H., et al. [69].	Invasion/migration
	PI3K/AKT	Wei, M., et al. [109]	Growth/metastasis
	CtBP2	Cui, T.X., et al. [19].	Sphere formation/ metastasis
Breast Cancer	EZH2	Jiang, H.B., et al. [110].	Proliferation/apoptosis
			Invasion/ migration
	Nrf2	Yi, J., et al. [35].	Proliferation/apoptosis
	Med19	Zhang, X.F., et al. [34].	Proliferation/migration /invasion
	Jak2	Wang, L., et al. [11].	Apoptosis
	COX-2-MMP1 signaling	Harati, et al. [70].	Metastasis
HER-2 positive Breast Cancer		Normann, et al. [92].	Drug sensitivity
Oral Cancer	BICC1	Wang, H., et al. [82].	Apoptosis
Colon Cancer	EZH2	Huang, Z.R., et al. [111].	Migration
	EZH2	Wang, G.Q., et al. [112].	Apoptosis
	STC1	Luan, C.Y., et al. [36].	Proliferation/migration /invasion
	Cox2	Cai, Y., et al. [113].	
	CREB1	Yang, Q.L., et al. [37].	Proliferation/migration
Colorectal Cancer	OGT/EZH2	Jiang, M.Z., et al. [114].	Metastasis
	Notch1	Wu, H.B., et al. [38].	Proliferation/metastasis
	ZEB1	Xiong, W.C., et al. [68].	Proliferation/migration
	HIPK3	Tao, L.P., et al. [115].	Drug resistance
	Rap1b	Zhou, Z.Y., et al. [67].	Progression
Pancreatic Cancer	STMN1	Zhu, L., et al. [39].	Proliferation/invasion
	autophagy	Zhang, X.F., et al. [94].	Drug resistance
Prostate Cancer	CUL4B	Gu, Z.H., et al. [48].	Proliferation/migrating /invasion
	Rap1A	Chen, J.H., et al. [40].	Proliferation/migration /invasion
Bladder Cancer	EZH2	Wang, K.P., et al. [116].	Proliferative/invasion /migration
	VEGF-C	Liu, P.H., et al. [117].	Drug resistance
	FZD4	Chen, L., et al. [71].	Migration/invasion
Papillary Thyroid Cancer	CLDN1	Du, Y.L., et al. [66].	Migration/invasion
	c-met and MCL-1	Zhu, J., et al. [87].	Apoptosis
Gastric Cancer	AMPK	Cao, S.G., et al. [26].	Proliferation
	EZH2	Cao, C., et al. [118].	Proliferation/migration and invasion
	PIM 1	Wu, F.B., et al. [25].	Proliferation/invasion /apoptosis
	SRF	Wu, X.Y., et al. [79].	Proliferation/invasion
Liver Cancer	ANXA2	Ma, S., et al. [45].	Proliferation
	MAPK/ERK pathway	Meng, X., et al. [22].	Proliferation
		Ding, A.K., et al. [119].	Proliferation/HBV replication
	HGF/c-Met pathway	Liu, Y., et al. [23].	Proliferation/migration
	ZNF217	Si, W.Z., et al. [24].	Proliferation/invasion
	VEGF-C	Liu, Z.Y., et al. [80].	Migration/invasion
	Girdin	Cao, K., et al. [81].	Proliferation/migration and invasion
Esophageal Squamous Cell Cancer	ATM and mTOR	Chen, M.Q., et al. [101].	Irradiation
	EZH2	Qiu, B.Q., et al. [120].	Proliferation/migration /invasion
Retinoblastoma	EZH2, HDAC9	Jin, Q.F., et al. [41].	Proliferation
Melanoma	RAP2B	Zhou, H., et al. [27].	Proliferation/migration/invasion
Glioblastoma	TRIM44	Li, L., et al. [72].	Proliferation/migration/invasion
Osteosarcoma	ZEB2	Lin, H.P., et al. [78].	Proliferation/invasion
